# End Point Surrogacy in First-Line Chronic Lymphocytic Leukemia

**DOI:** 10.1200/JCO.24.01192

**Published:** 2024-08-30

**Authors:** Florian Simon, Rudy Ligtvoet, Sandra Robrecht, Paula Cramer, Nadine Kutsch, Moritz Fürstenau, Valentin Goede, Julia von Tresckow, Petra Langerbeins, Anna-Maria Fink, Henriette Huber, Eugen Tausch, Christof Schneider, Clemens M. Wendtner, Matthias Ritgen, Martin Dreyling, Lothar Müller, Lutz Jacobasch, Werner J. Heinz, Ursula Vehling-Kaiser, Liliya Sivcheva, Sebastian Böttcher, Peter Dreger, Thomas Illmer, Michael Gregor, Philipp B. Staber, Stephan Stilgenbauer, Carsten U. Niemann, Arnon P. Kater, Kirsten Fischer, Barbara Eichhorst, Michael Hallek, Othman Al-Sawaf

**Affiliations:** ^1^ Faculty of Medicine and University Hospital Cologne, Department I of Internal Medicine, Center for Integrated Oncology Aachen Bonn Cologne Duesseldorf; German CLL Study Group, University of Cologne, Cologne, Germany; ^2^ St Marienhospital Cologne, Oncogeriatric Unit, Department of Geriatric Medicine, Cologne, Germany; ^3^ Clinic for Hematology and Stem Cell Transplantation, West German Cancer Center, University Hospital Essen, University of Duisburg-Essen, Essen, Germany; ^4^ Städtisches Klinikum Karlsruhe, Karlsruhe, Germany; ^5^ Department of Internal Medicine III, Division of CLL, Ulm University, Ulm, Germany; ^6^ Department of Medicine III, University Hospital, LMU Munich, Munich, Germany; ^7^ Department II of Internal Medicine, University of Schleswig-Holstein, Kiel, Germany; ^8^ Department of Medicine III, Ludwig-Maximilians-University Hospital, Munich, Germany; ^9^ Study Centrum Unter Ems, Practice for Oncology and Hematology, Leer, Germany; ^10^ Praxis of Haematology and Oncology, Dresden, Germany; ^11^ Caritas-Krankenhaus Bad Mergentheim, Medizinische Klinik II, Bad Mergentheim, Germany; ^12^ Outpatient Clinic, Landshut, Germany; ^13^ First Department of Internal Medicine, Multiprofile Hospital for Active Treatment – HristoBotev, Vratsa, Bulgaria; ^14^ Department of Medicine III Hematology, Oncology and Palliative Care, University Hospital, Rostock, Germany; ^15^ Department of Hematology, Oncology and Rheumatology, Heidelberg University Hospital, Heidelberg, Germany; ^16^ Division of Hematology, Cantonal Hospital of Lucerne, Lucerne, Switzerland; ^17^ Department of Medicine I, Division of Hematology & Hemostaseology, Medical University of Vienna, Vienna, Austria; ^18^ Department of Hematology, Copenhagen University Hospital—Rigshospitalet, Copenhagen, Denmark; ^19^ Academic Medical Department of Hematology, Cancer Center, Amsterdam, University of Amsterdam, Amsterdam, the Netherlands

## Abstract

**PURPOSE:**

Surrogate end points are commonly used to estimate treatment efficacy in clinical studies of chronic lymphocytic leukemia (CLL). This patient- and trial-level analysis describes the correlation between progression-free survival (PFS) and minimal residual disease (MRD) with overall survival (OS) in first-line trials for CLL.

**PATIENTS AND METHODS:**

First, patient-level correlation was confirmed using source data from 12 frontline German CLL Study Group (GCLLSG)-trials. Additionally, a joint-frailty copula model was fitted to validate correlation in the setting of targeted therapies (TT). Second, a meta-analysis of first-line phase III trials in CLL from 2008 to 2024 was performed. Treatment effect correlation was quantified from seven GCLLSG and nine published trials, using hazard ratios (HRs) for time-to-event and odds ratios for binary end points.

**RESULTS:**

The GCLLSG analysis set comprised 4,237 patients. Patient-level correlation for PFS/OS was strong with Spearman Rho >0.9. The joint-frailty copula indicated a weak correlation for chemotherapy/chemoimmunotherapy (C/CIT) with a tau of 0.52 (95% CI, 0.49 to 0.55) while the correlation was strong for TT (tau, 0.91 [95% CI, 0.89 to 0.93). The meta-analysis set contained a total of 8,065 patients including 5,198 (64%) patients treated with C/CIT and 2,867 (36%) treated with TT. Treatment-effect correlation of the HRs for PFS and OS was *R* = 0.75 (95% CI, 0.74 to 0.76, *R*^2^ = 0.56) while correlation of end-of-treatment MRD with PFS and OS was *R* = 0.88 (95% CI, –0.87 to 0.89; *R*^2^ = 0.78) and 0.71 (95% CI, 0.69 to 0.73; *R*^2^ = 0.5), respectively.

**CONCLUSION:**

Patient-level correlation was confirmed in the setting of TTs while treatment-effect correlation between PFS and OS remains uncertain. MRD response status showed a high treatment-effect correlation with PFS but not OS, with the caveat of a limited number of randomized trials with available MRD data.

## INTRODUCTION

Chronic lymphocytic leukemia (CLL), with 65 diagnoses per 100,000 people,^1^ the most prevalent leukemia in Europe and Northern America, is an indolent B-cell non-Hodgkin lymphoma and characterized by heterogeneous clinical and genetic properties.^2^ The advent of combination regimens including immunotherapeutic agents and more recently targeted therapies (TTs) has transformed the treatment landscape, with overall survival (OS) rates exceeding 7 years.^3-6^ OS is considered the most patient-relevant end point in oncology trials, but implementing it as a primary outcome measure in studies of CLL entails several challenges: The high burden of comorbidities in the CLL patient population poses a competing survival risk that can mask CLL-specific treatment effects. Moreover, the high efficacy of TTs warrants increasingly long observation periods or extensive patient recruitment, which can be operationally challenging. Finally, potent second- and third-line regimens can provide effective salvage options for relapsed CLL and thereby attenuate a potential OS advantage of a first-line regimen over prior standards of care. Hence, most clinical trials in CLL use surrogate and composite end points to retrieve information on treatment efficacy.^2^ However, the wide use of surrogate markers like progression-free survival (PFS), response rates, or minimal residual disease (MRD) in studies of CLL has been subject to criticism since the correlation between these surrogates and OS as the gold standard remains unclear.^7,8^ Only few CLL trials have demonstrated significant PFS and OS differences,^4-6^ while in many instances the reported PFS improvement so far did not result in significant OS advantages for the exploratory study arms.^9-12^ Therefore, data on the validation of PFS as a surrogate end point via a meta-analytic approach are urgently needed but so far lacking.

**CONTEXT**

**Key Objective**
This analysis aimed to analyze the patient- and trial-level correlation between progression-free survival (PFS), minimal residual disease (MRD) level, and overall survival (OS) in chronic lymphocytic leukemia (CLL), using trial and individual patient data of 8,065 patients. The objective was to investigate the validity of commonly used surrogate end points.
**Knowledge Generated**
A strong patient-level correlation between PFS and OS was observed while treatment-level correlations remained uncertain. Treatment-effect correlation of end-of-treatment MRD levels was strong for PFS, but not OS. Additional follow-up is needed to fully support their use as surrogates for OS.
**Relevance *(S. Lentzsch)***
Despite promising findings in this analysis, prospective studies will clarify the validity of PFS (and OS) surrogacy, with a focus on the potential of MRD as an integral biomarker in CLL treatment strategies. Given the clear PFS benefit in patients who achieve undetectable MRD after therapy, novel strategies incorporating MRD as an endpoint are ready for evaluation as a precision approach to CLL therapy.
*Relevance section written by *JCO* Associate Editor Suzanne Lentzsch, MD, PhD.

Several research articles have previously demonstrated a favorable correlation between features like PFS, response and minimal residual disease (MRD), and OS in the setting of CLL.^3,13,14^ Although the data supporting this correlation are promising, large-scale surrogacy studies incorporating treatment-effect analyses in the setting of diverse patient populations and treatment modalities are currently lacking.

The aim of this study was to evaluate the individual patient-level and treatment-level correlation between commonly used surrogate end points in CLL including PFS and MRD with OS and thereby provide an estimate on the adequacy of surrogate end points in first-line treatments for CLL. To accomplish this, individual patient data obtained from more than 4,000 treatment-naïve participants enrolled in 12 prospective clinical trials of the German CLL Study Group (GCLLSG) and published data from nine randomized phase three trials were analyzed.

## PATIENTS AND METHODS

### Patients and Studies

Twelve phase II and phase III trials of the GCLLSG evaluating time-limited therapies from 1999 to 2022 were analyzed.^15-27^ The included trials evaluated frontline treatment of CLL and enrolled patient cohorts of all age, fitness, and prognostic groups. All patients who were included in the safety analysis populations of the trials were considered for this analysis, as intention-to-treat analyses might be prone to imbalances because of differences in study design. Informed consent was obtained for all trial participants and each trial included in this study had obtained initial approval from local ethics committees.

Additionally, as treatment-effect evaluation of surrogate end points relies on large data sets and number of included trials, the MEDLINE database was searched for first-line, randomized phase III trials in CLL reported over the past 15 years. The search was conducted on April 30, 2024, by using key words CLL, small lymphocytic lymphoma in the title and first-line, untreated, newly diagnosed, treatment-naïve as title words starting from April 1, 2018. Inclusion criteria were multicenter randomized phase III trials in adult patients with active disease requiring treatment defined by international workshop on CLL (iwCLL) 2008 or 2018 criteria with reported/published values for hazard ratio (HR) PFS and HR OS. Excluded were reviews, observational trials, or trials evaluating supportive measures, trials in asymptomatic disease, and previously treated/relapsed/refractory disease. In case of multiple publications for one trial, only the most recent publication was evaluated.

### End Points and Criteria for Evaluation

For the GCLLSG data set, median observation time was calculated using all patients. PFS was calculated from random assignment/study inclusion to the date of progressive disease or death of any cause. OS was calculated from random assignment/study inclusion to the date of death from any cause. Patients were censored for PFS on the date of the last clinical/disease assessment and for OS at last seen alive.

Responses in all studies were investigator-assessed according to iwCLL guidelines.^28,29^ MRD measurements in the peripheral blood were assessed in reference laboratories via flow cytometry or allele specific oligonucleotide-polymerase chain reaction to a sensitivity of 10^−4^ that is, 1 in 10,000 cells.

MRD measurements were assessed at the end of treatment (for fixed-duration regimens) or end of induction treatment (for MRD-guided treatments; EO[I]T). EO(I)T ranged from month 9 to month 15, depending on the study protocol. In MRD-guided treatments, therapy with targeted agents for up to 36 months was possible.

### Statistical Analyses

**FIG 1.**
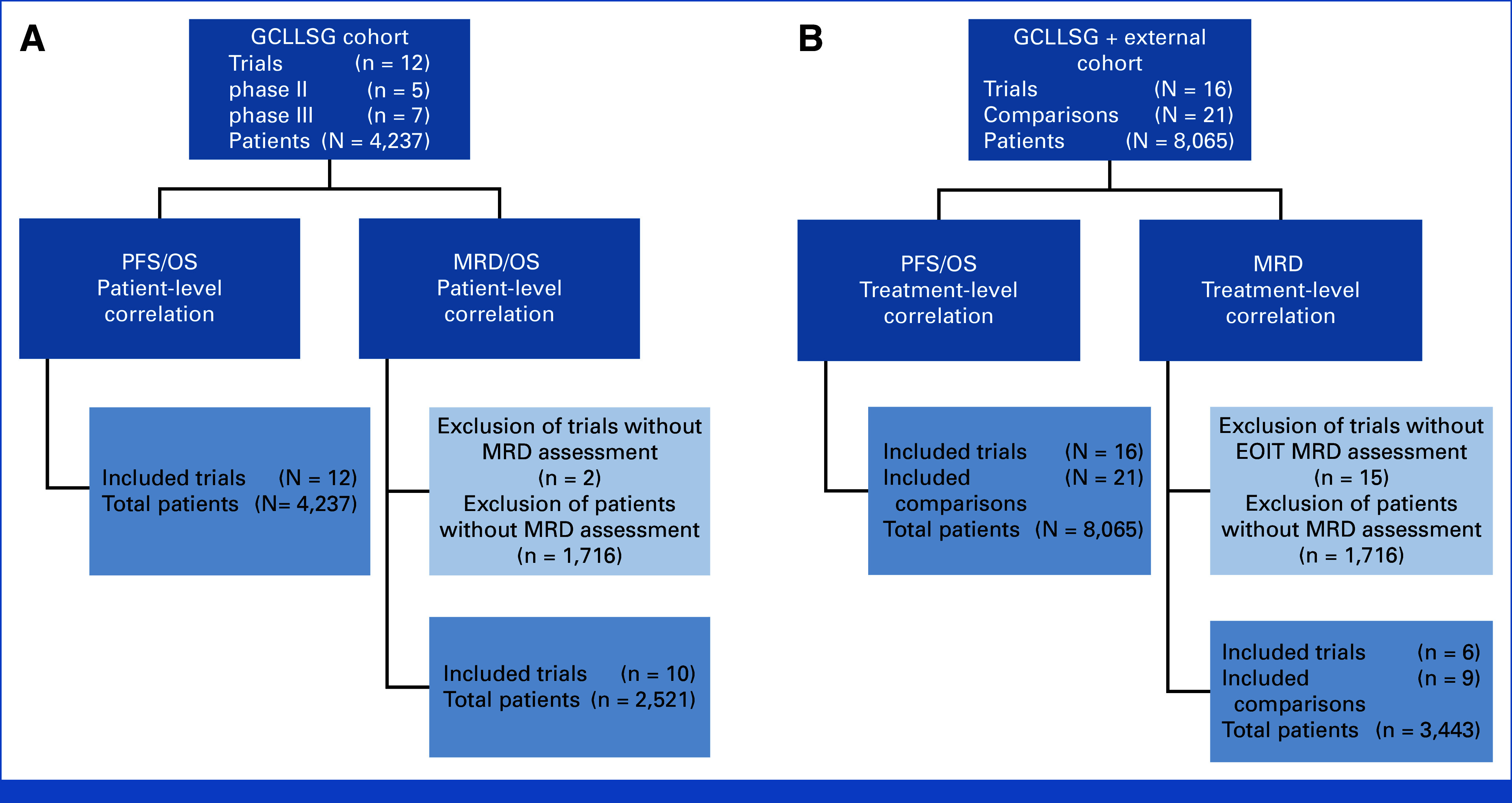
Flow chart of patient and trial selection. (A) Flow chart of patient and trial selection for patient-level correlation analyses. (B) Flow chart of patient and treatment comparisons for trial-level correlation analyses. EOIT, end of induction therapy; GCLLSG, German CLL Study Group; MRD, minimal residual disease; OS, overall survival; PFS, progression-free survival.

To determine validity of surrogate end points, we followed guidance laid out by the *Institut für Qualität und Wirtschaftlichkeit im Gesundheitswesen*^30^ (Institute für quality and economics in health care, IQWiG): first, individual patient-level correlation between the surrogate and true end point was established using source data from 12 GCLLSG trials (Fig 1A).

Treatment-effect correlation was then determined via a meta-analytic approach^31,32^ using combined data from randomized phase III GCLLSG and published trials (Fig 1B).

Treatment-effect correlation coefficient values ≤0.7 were considered as weak, >0.7 to <0.85 as moderate, and ≥0.85 as strong indicators for the validity of surrogate end points.^30^ The same thresholds were applied to individual-patient level correlation, although not formally established in the validity of surrogate end points.

### Patient-Level Surrogacy

To assess individual level surrogacy, Spearman's Rho was calculated using Kaplan-Meier functions estimated by using individual patient data: The probability of survival (OS and PFS) was estimated at different time points measured from random assignment/study inclusion. Estimated survival rates at these time points were then correlated across studies and weighted by the number of patients in each corresponding study using Spearman correlation coefficients, as shown previously by Ecker et al.^33^

To further confirm individual patient-level correlation in the setting of TTs the joint-frailty copula model was fitted to the data of GCLLSG phase III trials.^34,35^ Like Spearman Rho, Kendall's tau estimated with the joint-frailty copula takes on a value between –1 (perfect disagreement) and 1 (perfect agreement).

Additionally, PFS and OS according to MRD and iwCLL response status at EO(I)T were compared using Kaplan-Meier functions calculated from the time point of MRD or response assessment. For the PFS landmark analysis according to MRD and response status, patients with a progressive disease event at MRD assessment ±7 days were excluded.

### Trial-Level Surrogacy

Treatment effects in time-to-event end points were quantified by calculating HRs for OS and PFS in seven GCLLSG and nine published phase III trials evaluating first-line treatment in a total of 8,065 patients.

Treatment effects for binary end point MRD (detectable *v* nondetectable) were quantified by calculating odds ratios (ORs) at EO(I)T and compared with HRs for OS and PFS. In trials with multiple predetermined comparisons between treatment arms, such as CLL11, CLL13, or ELEVATE-TN, each HR/OR was accounted for as an individual treatment comparison.

Correlation was determined via Pearson *R*, weighted by the number of patients per trial comparison, and the coefficient of determination *R*^2^ for trial-level correlation. 95% CIs were based on Fisher r-to-z transformation.

For all analyses, *P* values of < .05 were determined to be significant. All analyses were done using SPSS (29.0.0.0 [241]) and R software (R Core Team 2022, Version 2.2.1, R Foundation for Statistical Computing, Vienna, Austria). An in-depth explanation of the employed methods and additional sensitivity analyses can be accessed in the supplemental material (Supplemental methods and analyses).

## RESULTS

### Patient Characteristics

#### GCLLSG Data Set

Overall, 4,237 patients from 12 study data sets received first-line CLL therapy and were included in the GCLLSG data set (Fig 1 and Data Supplement, Table S8). The median observation time was 67 months (range, 0-151). The median observation time was shorter for patients receiving TT compared with C/CIT with 41 versus 72 months (Data Supplement, Table S9).

The median age at study inclusion was 64 years (range, 27-90) with 45.1% of patients being older than 65 years (Table 1); 26.8% of patients had relevant comorbidities with a Cumulative Illness Rating Scale (CIRS)-score of >6. *TP53* mutation and/or del(17p) were prevalent in 347 (9.5%) of 3,652 evaluable patients, whereas 2,302 (61.6%) of 3,737 evaluable patients had an unmutated immunoglobuline heavy chain variable region. A total of 3,159 patients (74.6%) had received C/CIT, whereas 1,078 (25.4%) were treated with targeted combination therapies. One hundred thirty-six patients were treated beyond the end of induction therapy (EOIT) for a maximum of 36 months (Data Supplement, Table S10).

**TABLE 1.** Patient Characteristics GCLLSG-Data Set

Patient Characteristic	N = 4,237
Age at study inclusion, years	
Median	64
IQR	57-71
Range	27-90
Sex, No. (%)	
Female	1,281 (30.2)
Male	2,956 (69.8)
Age category at study inclusion, years, No. (%)	
≤65	2,327 (54.9)
>65	1,910 (45.1)
*TP53* mutation and/or del(17p), No. (%)	
Evaluable patients	3,652 (86.2)
Yes	347 (9.5)
No	3,305 (90.5)
Missing	585 (13.8)
IgHV mutational status, No. (%)	
Evaluable patients	3,737 (88.2)
Mutated	1,435 (38.4)
Unmutated	2,302 (61.6)
Missing	500 (11.8)
CLL-IPI risk category, No. (%)	
Low	793 (23.4)
Intermediate	1,672 (49.3)
High	699 (20.6)
Very high	228 (6.7)
Missing/not applicable	845 (19.9)
CIRS score category, No. (%)	
≤6	2,696 (73.2)
>6	986 (26.8)
Missing	555 (13.1)
Therapy category, No. (%)	
Chemoimmunotherapy^a^	3,159 (74.6)
Targeted therapy^b^	1,078 (25.4)
Second-line therapy by therapy	
Subsequent treatment after chemoimmunotherapy,^a^ No.	1,490
Chemoimmunotherapy, No. (%)	1,327 (89.1)
Targeted therapy, No. (%)	163 (10.9)
Subsequent treatment after targeted therapy,^b^ No.	107
Chemoimmunotherapy, No. (%)	38 (35.5)
Targeted therapy, No. (%)	69 (64.5)
No recorded subsequent therapy, No. (%)	2,640 (62.3)
Recorded PD and OS events, No. (%)	
None (neither PD nor death)	1,753 (41.4)
Only PD	1,273 (30)
Only death	370 (8.7)
PD before death	841 (19.8)
MRD at EO(I)T, No. (%)	
Total	2,521 (59.5)
Undetectable	1,489 (59.1)
Chemoimmunotherapy	697 (46.8)
Targeted therapy	792 (53.2)
Intermediate	546 (21.7)
Chemoimmunotherapy	407 (74.5)
Targeted therapy	139 (25.5)
High	486 (19.3)
Chemoimmunotherapy	435 (89.5)
Targeted therapy	51 (10.5)
Missing	1,716 (40.5)
Response at EO(I)T, No. (%)	
Total	3,807 (89.9)
Noncomplete response	2,607 (68.5)
Complete response	1,200 (31.5)
Missing	430 (10.1)

NOTE. MRD measured in the peripheral blood via flow cytometry. Undetectable: <10^−4^, intermediate: 10^−4^ to 10^−2^, high: ≥10^−2^.

Abbreviations: CIRS, cumulative illness rating scale; CLL, chronic lymphocytic leukemia; EO(I)T, end of (induction)treatment; IgHV, immunoglobuline heavy chain variable reagion; MRD, minimal residual disease; OS, overall survival; PD, progressive disease.

^a^ Fludarabine, chlorambucil mono, fludarabine + cyclophosphamide, fludarabine + cyclophosphamide + rituximab, bendamustine + rituximab, rituximab/obinutuzumab + chlorambucil treatment arm.

^b^ Rituximab + venetoclax, obinutuzumab + venetoclax, obinutuzumab + ibrutinib + venetoclax. Bendamustine + venetoclax + obinutuzumab, bendamustine + idelalisib + obintuzumab, bendamustine + ibrutinib + obinutuzumab or bendamustine + ibrutinib + ofatumumab.

Overall, 2,114 progressive disease (PD) events and 1,211 deaths were captured. The median PFS and OS across all trials was 44.8 months and 100.6 months, respectively (Data Supplement, Table S11).

A total of 1,597 patients received subsequent therapy, of whom 232 (14.5%) were treated with targeted agents and 1,365 (85.5%) with C/CIT approaches. Additional baseline characteristics are summarized in Table 1.

#### Meta-Analysis Data Set

For the meta-analysis, in addition to seven GCLLSG trials, nine trials were identified with a total of 13 reported treatment arm comparisons.^4,6,36-43^ The ALLIANCE trial was excluded because of missing data on HRs for OS. The external data set contained 10 treatment comparisons including 3,993 patients. The median age was between 62 and 72 years, and all trials included a majority of male patients. Additional baseline characteristics are summarized in Table 2.

**TABLE 2.** Patient Characteristics External Data Set

Study	No.	Study Arm	No.	Age, Years (range)	Male Sex, No. (%)	TP53 Ab, No. (%)	uIGHV, No. (%)	CIRS >6, No. (%)
RESONATE2	269	Experimental	136	Median 73 (65-89)	88 (65)	0	58 (43)	42 (31)
		Control	133	Median 72 (65-90)	81 (61)	0	60 (45)	44 (33)
ECOG	529	Experimental	354	Mean 56.7	236 (66.7)	2 (0.6)	210 (75)	NA
		Control	175	Mean 56.7	120 (68.6)	0	71 (61.7)	NA
FLAIR IR *v* FCR	771	Experimental	386	Median 63 (55-67)	283 (73)	2 (1)	194 (50)	NA
		Control	385	Median 62 (56-67)	282 (73)	1 (<1)	194 (50)	NA
FLAIR IV *v* FCR	523	Experimental	260	Median 62 (55-67)	186 (71.5)	1 (0.4)	123 (47.3)	NA
		Control	260	Median 62 (57-67)	181 (68.8)	0	138 (52.5)	NA
ELEVATE-TN	535	Experimental (A + O)	179	Median 70 (65-75)	111 (62)	25 (14)	103 (57.7)	30 (16.8)
		Experimental II (A)	179	Median 70 (66-75)	111 (62)	23 (12.8)	119 (66.5)	21 (11.7)
		Control	177	Median 71 (67-76)	106 (59.9)	25 (14.1)	116 (65.5)	15 (8.5)
SEQUOIA	479	Experimental	241	Median 70 (66-75)	154 (64)	2 (1)	125 (53)	NA
		Control	238	Median 70 (66-74)	144 (61)	0	121 (52)	NA
Illuminate	229	Experimental	113	Median 70 (66-75)	67 (59)	18 (16)	66 (62)	37 (33)
		Control	116	Median 72 (66-77)	79 (68)	23 (20)	57 (53)	36 (31)
GLOW	211	Experimental	106	Median 71 (47-93)	59 (55.7)	7 (6.6)	55 (51.9)	74 (69.8)
		Control	105	Median 71 (57-88)	63 (60)	2 (1.9)	54 (51.4)	61 (58.1)
COMPLEMENT-1	447	Experimental	221	Median 69 (35-92)	142 (64)	10 (5)	114 (57)	NA
		Control	226	Median 70 (36-91)	140 (62)	17 (8)	113 (56)	NA

Abbreviations: A, acalabrutinib; CIRS, Cumulative Illness Rating Scale; IgHV, IGHV: immunoglobuline heavy chain variable region; O, obinutuzumab; TP53ab, TP53 aberration; uIGHV, unmutated immunoglobuline heavy chain variable region.

Thus, 10 treatment comparisons from external studies and 11 treatment comparisons from GCLLSG trials were included into our analysis, leading to a total of 8,065 patients, of whom 5,198 (64%) patients were treated with C/CIT and 2,867 (36%) were treated with TT (Fig 1, Data Supplement, Table S12). All trials had PFS as their primary end point, except CLL4 and CLL5 which used OS.

### Patient-Level Surrogacy

#### Correlation Between PFS and OS

At 24/60 months, Spearman Rho was 0.966 (95% CI, 0.96 to 0.97; Fig 2A). With other estimates ranging from 0.92 to 0.97, a consistently high correlation was observed throughout all time points (Data Supplement, Figs S5-S19).

**FIG 2.**
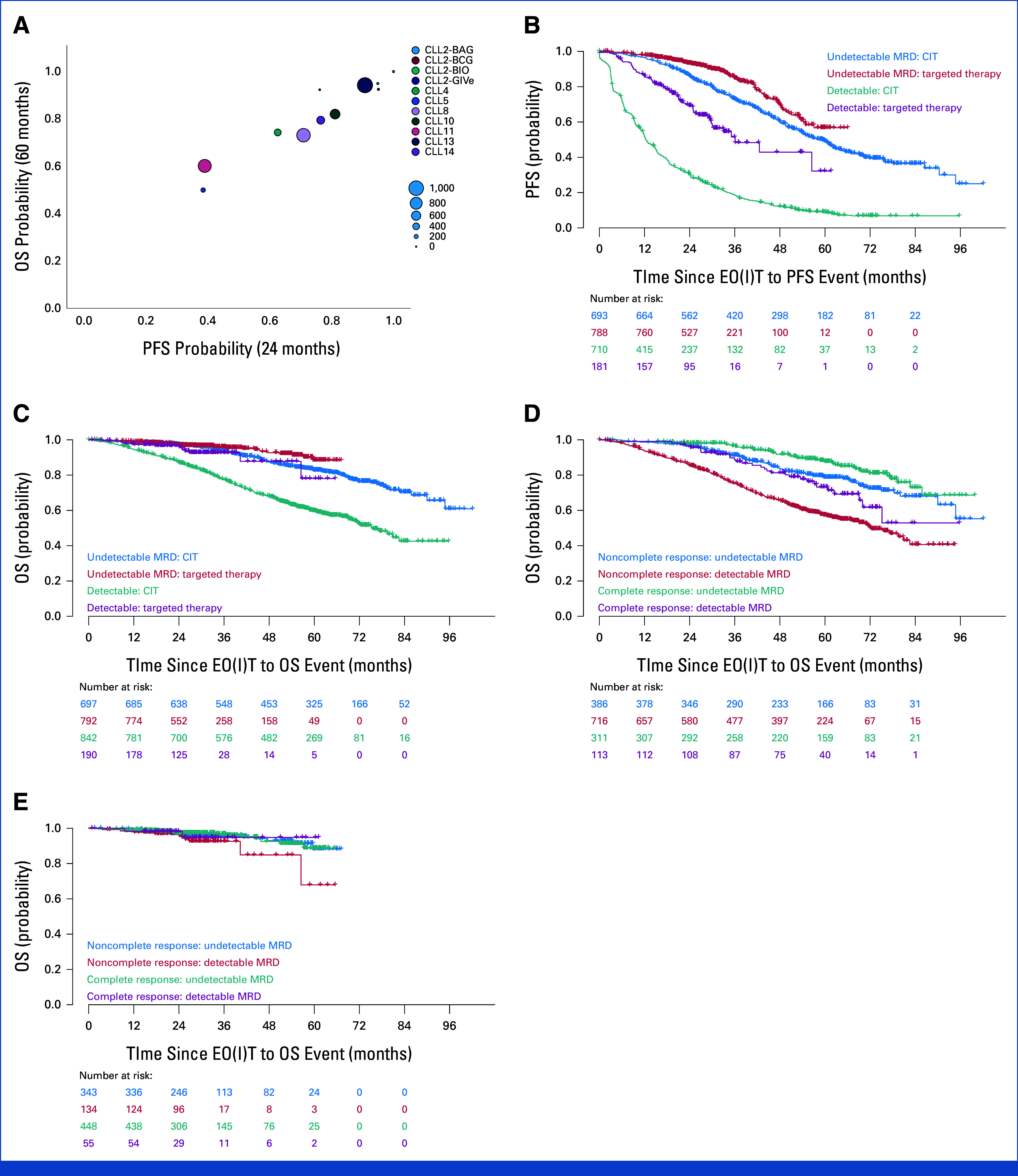
Patient-level surrogacy and correlation analyses. (A) Trial-level results for estimated PFS rate at 24 months (*x* axis) and OS rate at 60 months (*y* axis) are shown. The size of each dot corresponds with the number of included patients (n = 4,237). (B) PFS according to MRD status and type of treatment. MRD measured in peripheral blood via flow cytometry or ASO-PCR with a sensitivity of 10^−4^. Undetectable MRD: <10^−4^. Detectable MRD: ≥10^−4^. (C) OS according to MRD status and type of treatment. MRD measured in peripheral blood via flow cytometry or ASO-PCR with a sensitivity of 10^−4^. Undetectable MRD: <10^−4^. Detectable MRD: ≥10^−4^. (D) OS since EO(I)T by CR and MRD for chemoimmunotherapy. (E) OS since EO(I)T by CR and MRD for targeted agents. ASO-PCR, allele specific oligonucleotide-polymerase chain reaction; CIT, chemoimmunotherapy; CR, complete response; EOIT, end of induction therapy; MRD, minimal residual disease; OS, overall survival; PFS, progression-free survival.

Using the joint-frailty copula model, effects of TTs versus CIT across trials on both the PFS and OS hazard on the individual patient level were found (HR, 0.14 [95% CI, 0.07 to 0.25] and HR, 0.43 [95% CI, 0.35 to 0.53], respectively). For patients treated with CIT separately however, Kendall's tau estimated with the joint-frailty model of concordance showed only a moderate relationship between PFS and OS (tau, 0.52 [95% CI, 0.49 to 0.55]), whereas the correlation was strong for patients treated with TT with a tau of 0.91 (95% CI, 0.89 to 0.93).

Additional sensitivity analyses focused on time-to-event correlation confirmed the findings of an increased correlation of PFS and OS in patients treated with TT (Data Supplement, Tables S1-S4, Figs S1-S4).

#### Correlation Between MRD Status With PFS and OS

MRD measurements at EO(I)T were available for 2,521 patients, representing 59.5% of the study population. Rates of undetectable (<10^−4^), intermediate (≥10^−4^ and <10^−2^), and high MRD (≥10^−2^) were 59.1%, 21.7%, and 19.3%, respectively.

Detectable (ie, >10^−4^) versus undetectable MRD results influenced PFS with a median of 18.6 months versus 61.9 months and a HR of 4.74 (95% CI, 4.20 to 5.36, *P* < .001). This effect was also significant when differentiating by TT and C/CIT with HR of 3.15 (95% CI, 2.32 to 4.28, *P* < .001) and 4.28 (95% CI, 3.73 to 4.91, *P* < .001), respectively (Fig 2B). The median PFS was 42.5 months versus not reached and 15.3 versus 59.3 months, respectively, for TT and C/CIT.

In Cox regression analysis of detectable and undetectable MRD, the median OS was 77.1 months and not reached while 60-month OS rates were 61.4% and 84.6%, respectively. The HR for OS in detectable versus undetectable MRD was 3.02 (95% CI, 2.5 to 3.64, *P* < .001)

A detectable versus undetectable MRD level was associated with a worse OS for both TT and C/CIT with a HR of 1.96 (95% CI, 1.04 to 3.7, *P* = .038) and 2.7 (95% CI, 2.2 to 3.33, *P* < .001), respectively (Fig 2C).

When combining response and MRD status, there was a significant difference between OS for uMRD in complete versus noncomplete responders with a HR of 0.66 (95% CI, 0.48 to 0.91, *P* = .011). However, when differentiating by type of therapy, the OS difference was only significant in patients treated with CIT (Fig 2D), but not TT (Fig 2E) with HRs of 0.63 (95% CI, 0.44 to 0.91, *P* = .014) and 0.9 (95% CI, 0.46 to 1.74, *P* = .75), respectively.

### Trial-Level Surrogacy

#### Correlation Between PFS and OS

**FIG 3.**
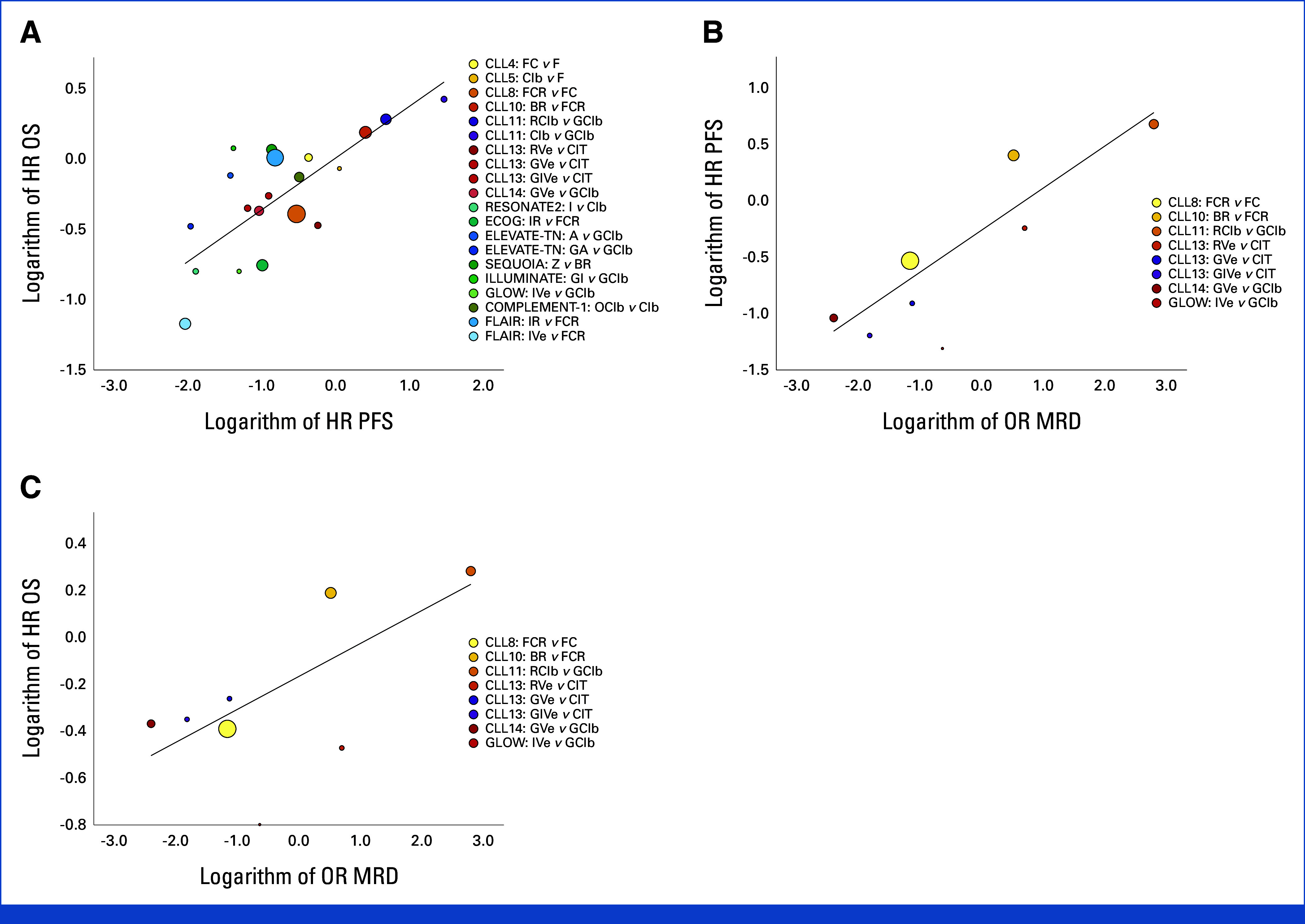
Trial-level surrogacy and correlation analysis. (A) Scatterplot of HR for PFS and OS. Each circle represents a different trial/trial comparison. Sizes of the circles correspond with the number of included patients. (B) Scatterplot of ORs for undetectable MRD and HR PFS. Each circle represents a different trial/trial comparison. Sizes of the circles correspond with the number of included patients. (C) Scatterplot of OR for undetectable MRD and HR OS. Each circle represents a different trial/trial comparison. Sizes of the circles correspond with the number of included patients. HR, hazard ratio; MRD, minimal residual disease; OR, odds ratio; OS, overall survival; PFS, progression-free survival.

The treatment-effect analysis showed a moderate trial-level correlation between HR for PFS and OS with a Pearson *R* = 0.75 (95% CI, 0.74 to 0.76, R^2^ = 0.56; Fig 3A), indicating that 56% of the variance in OS could be explained by differences in PFS between treatment arms.

#### Correlation Between MRD and PFS/OS

For treatment-effect correlation, only patients with MRD assessment at EOIT were considered. CLL4 and CLL5 were excluded as they did not perform MRD testing. The chlorambucil arm of CLL11 was excluded as no patient achieved MRD negativity. Additionally, the MRD data from the GLOW trial were included as this was the only other first-line trial outside the GCLLSG database with fixed-duration targeted treatment. Thus, six trials with a total of nine MRD comparisons and 3,443 patients were included for MRD treatment effect analyses.

A strong correlation was seen for MRD and PFS with *R* = 0.88 (95% CI, 0.87 to 0.89, *R*^*2*^ = 0.78; Fig 3B). The association with OS was moderate with *R* = 0.71 (95% CI, 0.69 to 0.73, *R*^*2*^ = 0.5; Fig 3C), limited by the number of events, especially in the setting of TTs. Treatment-effect correlation between MRD in BM at EOIT and PFS/OS showed similar results but was based on few data points (Data Supplement, Tables S5-S7).

## DISCUSSION

To our knowledge, this is the largest analysis of trial- and patient-level data exploring the use of surrogate end points in CLL. Additionally, this is the so far largest analysis of end-of-treatment MRD data and its prognostic impact. Individual patient-level correlations were strong and underline the prognostic significance of early PD and detectable MRD.

Treatment-effect correlation was moderate in the setting of PFS and OS; however, with *R* = 0.75 the validity of surrogacy remains unclear according to predefined thresholds.

The treatment-effect correlation between detectable MRD and PFS was strong, indicating a validity of surrogacy, whereas the correlation between MRD and OS was moderate; however, this was affected by a limited number of events, especially in patients with undetectable MRD, underlining the importance of longer follow-up in trials with incorporation of MRD measurements.

Previous analyses using the meta-analytic approach and individual patient data have been able to establish the use of PFS as a surrogate for OS in diffuse large B-cell lymphoma on the basis of a treatment-effect correlation of *R*^2^_WLS_ = 0.83.^44^ In follicular lymphoma, the 30-month complete response (CR) rate was suggested as a surrogate for PFS^45^; however, a recent report indicated weak correlation between PFS and OS with an *R* of only 0.38 and *R*^2^ = 0.15.^46^

No meta-analytic evaluation of surrogate end points has so far been performed in CLL; however, a previously performed literature-based correlation analysis of CLL trials found a patient-level correlation with a Spearman Rho of 0.81, which was mostly due to a strong correlation in the setting of subsequent-line therapies.^14^ Since only patients receiving first-line therapy have been included in this analysis, the applicability of the results in the relapsed/refractory disease setting is limited and with the advent of targeted therapeutic approaches, this correlation might have been distorted because of improved disease control over the past decade. However, when looking at the different therapy settings in our study, an improved correlation via joint-frailty copula modeling can be found for patients treated with targeted agents compared with CIT approaches. An exploratory analysis evaluating the time of study inclusion showed a higher patient-level correlation for patients with study inclusion after 2015 (data not shown) suggesting that the improved salvageability in subsequent lines and the up-front targeted therapy leads to this improved correlation.

MRD status assessed after EO(I)T with time-limited therapy was strongly associated with PFS and OS in this analysis. This result is in line with previous analyses, which were however based mostly on chemoimmunotherapeutic approaches^13,47,48^ or single-study analyses.^3,49^ Accordingly, recent trials involving time-limited targeted therapeutic approaches are incorporating MRD measurement as a (co)primary end point,^27^ in agreement with the EMA's decision to consider MRD as an intermediate end point.^50^ Whether treatment intensity and duration can be tailored on the basis of MRD status has been recently addressed in the randomized FLAIR study^51^ (EudraCT 2013-001944-76), and other randomized studies are exploring further MRD-guided strategies, such as MAJIC (ClinicalTrials.gov identifier: NCT05057494) and CLL18.

The iwCLL 2018 guidelines require a computed tomography (CT) scan and bone marrow biopsy to confirm CR in clinical trials.^29^ In line with previous findings,^13,52^ the CR status was not associated with survival in patients with undetectable MRD after targeted therapy. Future guidelines and regulators should take these findings into account, especially given the procedural disadvantages of CT scans and bone marrow biopsies compared with MRD measurements from peripheral blood.

Our study provides additional validation of the value of PFS as a surrogate end point in CLL, particularly in the current era of targeted agents and thereby confirms the regulators' strategy for drug approvals in CLL.^50,53^ Moreover, while the treatment-effect analysis of MRD status suggested a moderate correlation with OS, an improved correlation was observed with PFS, indicating that sample size and observation time might be too limited to elucidate associations with OS.

Surrogacy evaluation has so far not been standardized, and large-scale analyses such as this are needed for each indication/treatment setting and can therefore support recent efforts of multinational collaborations of key health care players such as NICE^54^ in determining cost-effectiveness of novel therapies, ultimately ensuring timely patient access.

Some limitations of this study need to be considered. Various methods for determining surrogacy have been previously suggested^30,31,55-58^; however, there is currently no formal guideline that provides a consensus on the best validated methodology. In this study, a strong individual patient-level correlation, between PFS and OS above >0.8, can be seen. However, the use of individual patient-level correlation as the sole method of validation was previously criticized^55,56^ and indeed the treatment-effect level analysis suggested a more modest surrogacy of PFS with OS. Although use of these two-level validation methods is widely established, limitations remain, especially with regards to inadequate reflection of censoring, which here were addressed by implementing the joint-frailty copula model, as well as weaknesses in the setting of low number of included trials.^34^

This analysis focused on patients undergoing first-line treatment and thus further independent validation in cohorts with relapsed/refractory CLL is warranted, with one meta-analysis previously indicating a correlation between PFS and OS also in the relapsed/refractory setting.^14^ Additionally, the number of patients requiring second-line treatment after first-line targeted therapy was low, so that insights into optimal treatment sequencing and its implications for surrogacy require longer observation periods. Finally, although PFS/OS correlation was based on results involving patients treated with both BTKi and venetoclax combinations, the treatment-effect analyses for MRD as a surrogate end point were based solely on end of treatment MRD levels.

In conclusion, this analysis demonstrates a strong individual-level correlation between PFS and OS and a moderate treatment-effect correlation. According to predefined and well-established thresholds, the validity of surrogacy for PFS and OS remains unclear underlining the need for further studies and especially large collaborative efforts to further elucidate the promising findings of this study. The survival benefit of patients with undetectable versus detectable MRD levels across all limited-duration treatment modalities and a strong treatment-effect correlation with PFS and moderate correlation with OS, reassures recent efforts to evaluate MRD-guided strategies to further optimize the treatment of CLL. Long-term follow-up of CLL trials is, however, still vital to validate a possible surrogacy of MRD for PFS and OS.

## Data Availability

The German CLL Study Group (GCLLSG) will consider academic data sharing requests on a case-by-case basis. The data will be released to such requesters with necessary agreements to enforce terms such as security, patient privacy, and consent of specified data use, consistent with evolving, applicable data protection laws. Data requests should be forwarded to the corresponding authors.
